# Counting, Cardinality and Conservation in Autistic Children

**DOI:** 10.1002/aur.70323

**Published:** 2026-07-23

**Authors:** Irene Polo‐Blanco, Raúl Fernández‐Cobos, Agustín Vicente

**Affiliations:** ^1^ Department of Mathematics, Statistics and Computer Science Universidad de Cantabria Santander Spain; ^2^ Department of Linguistics and Basque Studies Universidad del País Vasco (UPV/EHU) Vitoria‐Gasteiz Spain; ^3^ Ikerbasque—Basque Foundation for Science Bilbao Spain

**Keywords:** autism, cardinality, conservation, counting, equinumerosity, subitization

## Abstract

This study examined the understanding of cardinality and conservation in children with and without autism (aged 4–7 years, without intellectual disability), and its relation to counting strategies. A total of 82 children participated in the study, including 41 autistic children (36 boys, 5 girls) and 41 age‐ and sex‐matched non‐autistic peers. While both groups demonstrated similar mastery of cardinality, autistic children showed lower performance in conservation tasks. Response times were longer in autistic children during counting tasks. Concerning counting strategies, autistic children relied more on verbal counting with pointing (VCP) and used the rapid recognition strategy (N) less frequently, even for small canonical sets. While both strategies (N and VCP) supported cardinality in both autistic and non‐autistic children, only the N strategy appeared closely linked to successful conservation performance. These findings highlight two areas of relative difficulty for some autistic children and point to the potential of targeted interventions to strengthen foundational numerical skills and support later mathematical development.

## Summary

Some autistic children rely heavily on counting strategies that non‐autistic children employ less often (e.g., verbal counting while pointing, even for small numbers). We wondered whether such ways of counting could affect two milestones in mathematical development: cardinality (knowing that the last number counted represents the total number of items in a set) and conservation (realizing that the number of items stays the same even when the arrangement changes). In our study, autistic children aged 4–7 years used rapid, nonverbal counting less often than their non‐autistic peers of the same age, and those who did use this strategy performed better on conservation tasks. These findings highlight two potential areas for intervention that could help autistic children strengthen early numerical skills and support later mathematics learning.

## 1 Introduction

Typically developing (TD) children's understanding of number words and counting develops through a predictable series of conceptual stages (Carey 2009; Fuson 1988; Wynn 1990). Their journey typically begins around the age of two, when they first learn to recite the count list. At this point, the sequence of number words functions much like a memorized rhyme, fluid in performance but devoid of real numerical meaning. Gradually, children begin to uncover what individual number words refer to. This process is slow and effortful and typically unfolds between approximately 2.5 and 4 years of age: each of the early number words—“one,” “two,” “three,” and sometimes “four”—is acquired over several months of focused learning. During this period, TD children can correctly identify the quantity associated with only the number words they have mastered.

A striking cognitive shift occurs after children grasp the meanings of these first few number words. Rather than continuing to learn each subsequent number one by one, children appear to make an intuitive leap. Almost suddenly, they come to understand the general logic of the count sequence: that each successive word refers to a set that is exactly one larger than the previous. This realization allows them to determine the size of any set for which they know the corresponding count word. Children who have reached this point are known as full counters, and this transition represents a major milestone in numerical development (Carey 2009; Condry and Spelke 2008; Piantadosi et al. 2012; Sarnecka and Lee 2009; Wynn 1990, 1992). Typical ways of testing the cardinality principle are the *give me N* task (Wynn 1990, 1992) or just asking *how many Xs are there*? (Baroody et al. 2023).

Sarnecka and Wright (2013) suggest that by the time children understand cardinality, they also have some understanding of equinumerosity, that is, that all and only sets whose members can be placed in perfect one‐to‐one correspondence have the same number of items. However, children only pass equinumerosity tasks when they are put in a concrete way (e.g., *There are five flowers. Are there five vases or six?*). Yet, equinumerosity is typically assumed to relate to the next milestone in children's development of mathematical knowledge: conservation of number. This refers to the ability to understand that two sets remain equinumerous despite changes in spatial arrangement or other transformations (Piaget 1952), a concept that is generally mastered later, around 5 to 7 years of age. In parallel, a more primitive system for representing quantity, the approximate number system (ANS: Odic and Starr 2018), is present from infancy and allows children to discriminate between sets on the basis of numerical ratios before mastering exact number concepts. The precision of this system improves gradually across childhood, suggesting that approximate representations of quantity may provide an early, non‐symbolic foundation for later‐developing concepts such as cardinality and exact equality. In the case of autism, Chen et al. (2026) found that in TD children, ANS acuity was associated only with informal mathematical skills, whereas in children with ASD it was related to both informal and formal mathematical abilities.

Research on mathematical abilities in autism shows a heterogeneous developmental pattern, with relatively intact early skills but increasing variability over time (Aagten‐Murphy et al. 2015; Fernández‐Cobos and Polo‐Blanco 2024; Miller et al. 2017; Titeca et al. 2014, 2015, 2017; Wei et al. 2015). In preschool, several studies report that autistic children without intellectual disability perform comparably to TD peers across foundational numeracy tasks, including counting, magnitude comparison, estimation, and early arithmetic (Titeca et al. 2014, 2015, 2017; but see Fernández‐Cobos et al. 2026). However, from school age onward, differences become more apparent and variable: autistic children may show weaker performance in symbolic and non‐symbolic magnitude estimation (Aagten‐Murphy et al. 2015) and reduced precision in approximate number processing even after controlling for cognitive and language abilities (Li et al. 2023). Profile‐based studies reveal substantial heterogeneity, with some autistic individuals displaying relative mathematical strengths and others marked difficulties (Chen et al. 2019; Wei et al. 2015), and recent work suggests that group differences may become more pronounced with age across both formal and informal domains (Fernández‐Cobos and Polo‐Blanco 2024).

While the developmental progression of TD children ‐ from initially reciting the number list (often with errors) to mapping number words onto objects and ultimately grasping the cardinality principle‐ is well documented, much less is known about the specific counting strategies children use and how these strategies relate to their understanding of cardinality and numerical conservation. Numerous studies have documented TD children's spontaneous use of gesture while counting objects (Gelman and Gallistel 1978; Alibali and DiRusso 1999; Graham 1999; Taruscia et al. 2025). Children seem to have a tendency to either point, touch, or move objects as they count them. Although most researchers agree that gesture plays some role in facilitating counting, they disagree on the specific role that gesture plays in children's counting. Two possible functions of gesture in children's counting include keeping track of what has been counted and coordinating number words and the objects to be counted. Taruscia et al. (2025) observe that pointing gestures offer immediate benefits for children with weaker inhibition, but over time they provide greater gains for children with stronger working‐memory abilities as they build cardinality knowledge.

Although it is generally assumed that most children use both pointing gestures and number words while acquiring the cardinality principle, individual differences in counting behavior have not been systematically examined. Yet such differences may well exist, given the individual variation observed in other domains of gesture production (Congdon et al. 2025). In practice, children may engage in counting in at least the following ways: *verbal counting with pointing (VCP) or without pointing (VC)*, in which they recite number words while pointing to objects or without accompanying gestures, respectively; *pointing without verbal counting (P)*, in which children point to each object before producing the set's cardinality; and simply *stating the cardinality* without neither verbal counting or pointing *(N)*. Counting without overt counting behavior can be an internalization of verbal counting (i.e., counting, but in inner speech), or can involve another, very different way of counting: subitizing; that is, the ability to immediately and effortlessly recognize the number of items in a small set *without* counting them one by one (Wender and Rothkegel 2000). These variations reflect meaningful differences in how children coordinate verbal and non‐verbal components of counting, and may offer valuable insights into the processes that underlie their developing numerical concepts (e.g., subitizing can be a good predictor of cardinality: Splinter et al. 2025).

Our interest in different counting strategies stems from a broader aim to understand early mathematical abilities in autistic children (Aagten‐Murphy et al. 2015; Fernández‐Cobos and Polo‐Blanco 2024; Fernández‐Cobos et al. 2026; Miller et al. 2017; Titeca et al. 2014, 2015, 2017; Wei et al. 2015). Anecdotal observations suggest that autistic children tend to rely more heavily on VCP than TD children. In many cases, autistic children's pointing involves touching the objects directly, a pattern that may be related to well‐documented differences in pointing behaviors in autism (Ramos‐Cabo et al. 2021). It is therefore plausible that autistic children might be more likely to adhere to the first counting strategy they acquire, consistent with descriptions of insistence on sameness (Kanner 1943), potentially heightened experiences of uncertainty (Lawson et al. 2017) or maybe related to weaker executive functions (Taruscia et al. 2025). At the same time, autistic children may encounter greater difficulty with subitizing, particularly when items are arranged in canonical configurations –in non‐canonical configurations, also TD seem to exhibit difficulties (Jarrold and Russell 1997; Titeca et al. 2015). This challenge may reflect their characteristic enhanced local processing, which can make it harder to perceive grouped or global numerical patterns (Jarrold and Russell 1997).

In this paper we set out to explore the understanding of cardinality and of numerical conservation in children with and without autism (aged 4–7, without intellectual disability), and how they may relate to different counting strategies, within a developmental window characterized by rapid changes in early numerical competencies. By examining cardinality and conservation in parallel, we aim to determine whether autistic children follow similar, delayed, or distinct developmental pathways compared to their typically developing peers. By examining potential differences in successful counting strategies between the groups, we aim to connect children's methods of counting with their conceptual numerical knowledge. We also look into differences in response times and accuracy in counting tasks other than cardinality and conservation.

In sum, we set out to:

1. Examine the understanding of cardinality and conservation in children with and without autism.
2. Investigate potential differences between the autistic and non‐autistic groups in counting‐related variables, including response time, success rates, and counting strategies.
3. Explore which counting strategies correlate with performance and response times on a counting test, as well as with mastering cardinality and conservation.

We hypothesized that autistic children will rely more on verbal counting and pointing (VCP), often touching objects, making less use of rapid, non‐verbal strategies (N). This should affect their reaction times, but not necessarily accuracy. However, when it comes to the cardinality principle and conservation, we hypothesize that sticking to VCP may impact performance on cardinality and conservation. One reason relates to subitization, or at any rate, to rapid recognition of configurations of items, which has been suggested to be a foundational precursor of cardinality (Baroody and Lai 2022). Another reason relates to the hypothesized heavier use of VCP strategies, which, while they help avoid losing track, may focus attention on procedure rather than on the meaning of such a procedure.

In principle, we also would expect that the different counting strategies will be associated with cardinality and conservation in the same way in the two groups. That is, we would not expect that for example, VCP or P (just pointing) would strongly relate to success in cardinality and conservation in the autistic group but not in the non‐autistic group. However, we admit that this is a purely tentative hypothesis, as it may be that autistic individuals display a different developmental trajectory such that strategies that tend to be less successful than others in typical development turn out to be highly successful for autistic children.

Independently of counting strategies, we think we can expect inter‐group differences in tasks that cannot be solved by counting alone, such as conservation tasks, in which children must mentally represent two sets and establish one‐to‐one correspondences between their elements. These tasks appear to recruit executive functions (Houdé et al. 2011; Watanabe 2021, 2022), which are known to present challenges for many autistic children.

## 2 Methods

### 2.1 Participants and Setting

The study included 82 children with a mean age of 6.15 years (SD = 1.05; range = 4.2–7.8): 41 children with autism (36 boys and 5 girls) and 41 children without autism, matched by age and sex.

The autistic children were drawn from a larger ongoing project, recruited through child and adolescent mental health services and early intervention programmes. At the time of assessment, all participants were enrolled in mainstream schools within the Spanish educational system, where they followed the same national curriculum as their typically developing peers. The inclusion criteria for the autistic group were: (1) a diagnosis of Autism Spectrum Disorder (ASD) without additional psychiatric comorbidities according to the *Diagnostic and Statistical Manual of Mental Disorders* (DSM‐5; American Psychiatric Association 2013); (2) a non‐verbal IQ (NVIQ) of 70 or higher, assessed using the Leiter‐3 Nonverbal Intelligence Scale, administered entirely without verbal instructions or requiring verbal responses from participants (Koch et al. 2019; Roid and Miller 2013); (3) age between 4 and 7 years; and (4) the ability to understand brief instructions and point to stimuli. A child psychiatrist reviewed clinical records to confirm ASD diagnoses and the absence of additional psychiatric conditions, based on parent interviews and clinical evaluations.

Non‐autistic children were recruited from two local schools. A structured parent questionnaire was used as a screening tool to identify any neurological, developmental, or learning conditions that could affect participation in the study. The questionnaire included items on neurological diagnoses, biomedical conditions commonly associated with genetic or neurodevelopmental disorders (such as ASD, intellectual disability, or Down/William syndrome), hearing or vision impairments, oral‐motor speech difficulties, seizure disorders, ongoing interventions for communication or language, and difficulties with social interaction or participation in a variety of activities. Only children without any of these conditions were included as controls.

Children in the non‐autistic group were then matched by age and sex to those in the autism group, resulting in 41 matched pairs. NVIQ data were not available for the non‐autistic group. After receiving an explanation of the study, parents or legal guardians of children from both groups provided informed consent by signing the corresponding forms. No participants dropped out of the study. The study protocol was approved by the Ethics Committee for Clinical Research of Cantabria (CEIC‐C) and by the Ethics Committee for Research Involving Human Beings of the University of the Basque Country (CEISH).

Between two and three sessions were conducted with each participant. For the autistic group, sessions were carried out at the Faculty of Science of the University of Cantabria or at the Micaela Portilla Research Center in Vitoria, where participants also completed additional assessments as part of a larger ongoing project. Given the recruitment procedure and the dispersion of participants across a large number of schools, school‐based testing was unfeasible. In contrast, participants in the non‐autistic group were assessed in their respective schools, as this was the most feasible arrangement for data collection.

All sessions were conducted by the same experimenter in a distraction‐free classroom and were video‐recorded. The experimenter later reviewed the videos to code children's spontaneous counting strategies and accuracy, while response times were extracted using Audacity software. Response time (RT) was defined as the interval between the onset of the task instruction and the child's spontaneous response, with the last response considered in cases where the child changed their answer.

### 2.2 Measurement Variables and Instruments

#### 2.2.1 Counting Strategies

Counting strategies were evaluated with an adapted test based on Jarrold and Russell (1997) (*counting test*, from now on), which contained 15 cards with 4–8 black circles each. The cards were arranged in three configurations: (1) canonical patterns, such as familiar dice arrangements; (2) non‐canonical, with a random distribution of items; and (3) with distractors, interspersed non‐countable empty squares. Cards were presented in random order, and children were asked to count the circles.

#### 2.2.2 Cardinality and Conservation

For the evaluation of cardinality and conservation, several items from the TEMA‐3 (Test of Early Mathematics Ability; Ginsburg et al. 2007; Ginsburg and Baroody 2003) were considered. TEMA‐3 is a performance‐based instrument designed for children aged 3–9. The TEMA‐3 comprises 72 items evaluating both formal (31 items) and informal (41 items) mathematical skills. It has demonstrated high internal consistency, with a reported coefficient of 0.90 in typically developing populations (Ginsburg and Baroody 2003), and has been widely used in research with children with intellectual and developmental disabilities (e.g., Vostanis et al. 2021) as well as with children with autism (Fernández‐Cobos and Polo‐Blanco 2024; Polo‐Blanco et al. 2024).

Cardinality was assessed using a combination of measures of equal weight: TEMA‐3 items 7, 10, and 12, along with evidence from the counting test. In item 7, children counted a small set of stars and, after counting, were asked again, “How many stars did you count?” Item 10 required children to lift the correct number of fingers to represent a given quantity. Item 12 was assessed using the “give me *N* task,” in which children were asked to create a set containing a specified number of elements. Cardinality was also inferred from the adapted counting test: if children counted spontaneously without verbal recitation, cardinality was considered evident. If they recited the number sequence, the examiner asked at the end, “So how many are there?” If the child counted again from the beginning, cardinality was not evidenced; if the child stated the last number previously recited, cardinality was considered evidenced (last‐number rule; Fuson 1988).

Conservation was assessed using TEMA‐3 items 5, 8, and 11 (Ginsburg et al. 2007; Ginsburg and Baroody 2003), which evaluate early numerical competencies related to quantity equivalence and invariance across different task demands. Item 5 requires children to replicate the arrangement of 1 to 4 elements shown by the examiner, reflecting equinumerosity based on one‐to‐one correspondence. Item 8 requires children to adjust sets of items to match a target sum or difference under conditions of addition or subtraction and partial occlusion, involving the maintenance of numerical equivalence across transformations. Item 11 requires children to indicate the number of items in a set after perceptual rearrangement, assessing conservation of quantity, that is, the understanding that number remains invariant despite changes in spatial configuration.

Items 5 and 8 were intended to assess prerequisite competencies for conservation, specifically early equinumerosity and non‐symbolic numerical equivalence processes (both involving the instruction “place the same number of items as I do”), whereas Item 11 assesses conservation in the classical Piagetian sense (i.e., “I will count some items, then move them. After that, you will tell me how many there are without counting”). This interpretation is consistent with developmental accounts of set equivalence and one‐to‐one correspondence, and with evidence that young children can reason about quantity‐preserving versus quantity‐changing transformations, including hidden addition and subtraction operations, as part of early numerical reasoning prior to full conservation of quantity (Baroody 1987).

### 2.3 Data Analysis

Cardinality and conservation direct measures are categorical (yes/no, coded as 1/0). A cardinality score for each participant was calculated as the mean of the scores from items 7, 10, and 12 of the TEMA‐3, as well as a global score derived from the counting test. This global score was computed as the mean of the cardinality scores across the 15 cards of the test. The resulting cardinality score per participant is interpreted as the degree of acquisition of the cardinality principle. Similarly, a conservation score for each participant was computed as the mean of the scores from items 5, 8, and 11 of the TEMA‐3. Group comparisons were conducted for cardinality and conservation scores. To account potential influence of age, as well as possible differences in how these variables vary as a function of age across groups, linear models including an interaction term between group and mean‐centered age were fitted.

Comparisons between groups were also performed in the same manner for the counting test variables. Specifically, accuracy and RT were averaged across counting cards. In addition, RT differences across canonical, non‐canonical, and with‐distractor arrangements were analyzed within each group to replicate the study by Jarrold and Russell (1997). Statistical analyses were performed using linear mixed‐effects models, in which participant was included as a random effect to account for within‐subject dependencies.

Comparison of strategies between the autistic and non‐autistic groups was conducted using each participant's modal strategy across counting test cards, defined as the strategy used most frequently by each participant across trials. Binary logistic regression analyses with interaction term were conducted adjusting for centered age. Additionally, the use of these strategies for each counting test card was analyzed calculating the frequency of use within each group.

Developmental effects were assessed by computing Spearman's correlation tests between participants' age and the other measures within each group. Additionally, to explore potential relationships between the strategy choice and the other variables, Spearman's partial correlation tests were conducted within each group, controlling for age. Finally, analyses of covariance (ANCOVAs) were performed to examine whether preference for different strategies influenced accuracy and RT averaged across counting test cards, as well as performance on the cardinality and conservation scores. Centered age was included as a covariate to control for age‐related effects.

## 3 Results

The NVIQ in the autistic group had a mean of 98.15 (SE = 13.67; range = 71–131). A Kolmogorov–Smirnov test (*D* = 0.17; *p* = 0.15) indicated that the distribution was consistent with a normal distribution *N* (100, 15).

Table 1 presents the group comparisons for cardinality, conservation, accuracy, and RT, including the results of fitting linear models including group, centered age, and their interaction. There was a significant main effect of group in terms of conservation and RT averaged across counting test cards. Specifically, the autistic group showed lower conservation scores (β=−0.18, SE=0.06, p=0.003) and higher RT (β=2.27,SE=0.57,p<0.001) than the non‐autistic group at the mean age. The effect of age was statistically significant for cardinality, conservation, and accuracy, but not for RT. The interaction between group and age did not reach statistical significance for any variable; however, a trend was observed for accuracy (β=−0.07, SE=0.04, p=0.072) and RT (β=−1.05, SE=0.56, p=0.065), suggesting a possible difference between groups in developmental trajectories, with a less steep increase in accuracy and steeper decrease in RT with age in the autistic group.

**TABLE 1:** Group comparisons for cardinality, conservation, accuracy, and response times.

		(SE)	*p*	95% CI
Cardinality	Intercept:	0.94 (0.03)	< 0.001***	[0.89, 1.00]
	Group (ASD):	−0.06 (0.04)	0.141	[−0.13, 0.02]
	Centered age:	0.08 (0.03)	0.004**	[0.03, 0.13]
	Group × Age:	0.04 (0.04)	0.570	[−0.09, 0.05]
Conservation	Intercept:	0.90 (0.04)	< 0.001***	[0.82, 0.99]
	Group (ASD):	−0.18 (0.06)	0.003**	[−0.30, −0.06]
	Centered age:	0.13 (0.04)	0.002**	[0.05, 0.21]
	Group × Age:	−0.04 (0.06)	0.540	[−0.15, 0.08]
Mean accuracy	Intercept:	0.90 (0.03)	< 0.001***	[0.85, 0.95]
	Group (ASD):	−0.03 (0.04)	0.367	[−0.11, 0.04]
	Centered age:	0.10 (0.03)	< 0.001***	[0.04, 0.15]
	Group × Age:	−0.07 (0.04)	0.072	[−0.14, 0.01]
Mean RT	Intercept:	2.93 (0.40)	< 0.001***	[2.14, 3.74]
	Group (ASD):	2.27 (0.57)	< 0.001***	[1.14, 3.41]
	Centered age:	−0.52 (0.40)	0.196	[−1.30, 0.27]
	Group × Age:	−1.05 (0.56)	0.065	[−2.16, 0.07]

*Note:* * < 0.05, ** < 0.01, ***≤ 0.001.

**FIGURE 1:**
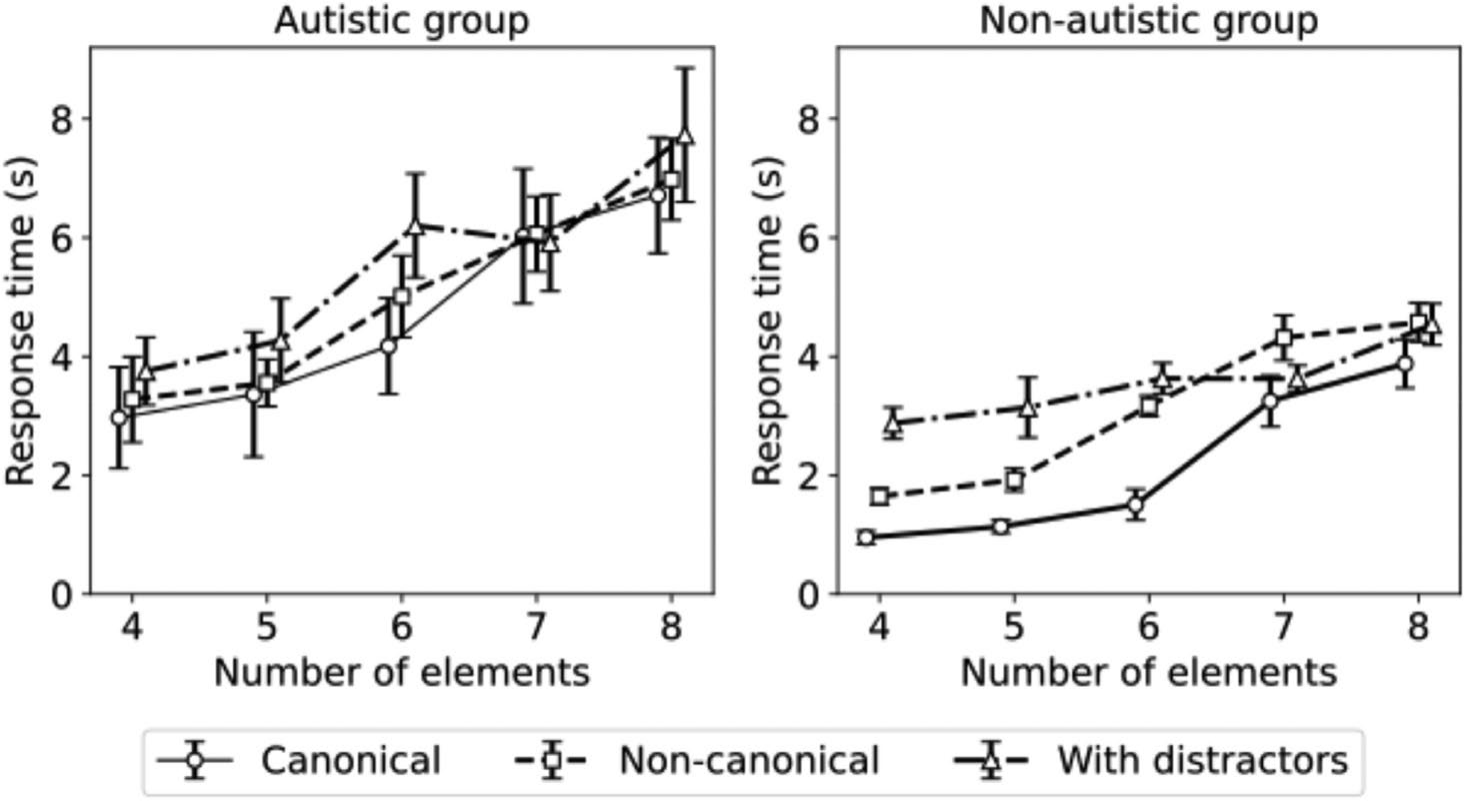
Response times as a function of the number of elements for different configurations. Response times for the different series were slightly offset along the x‐axis for visualization purposes.

**FIGURE 2:**
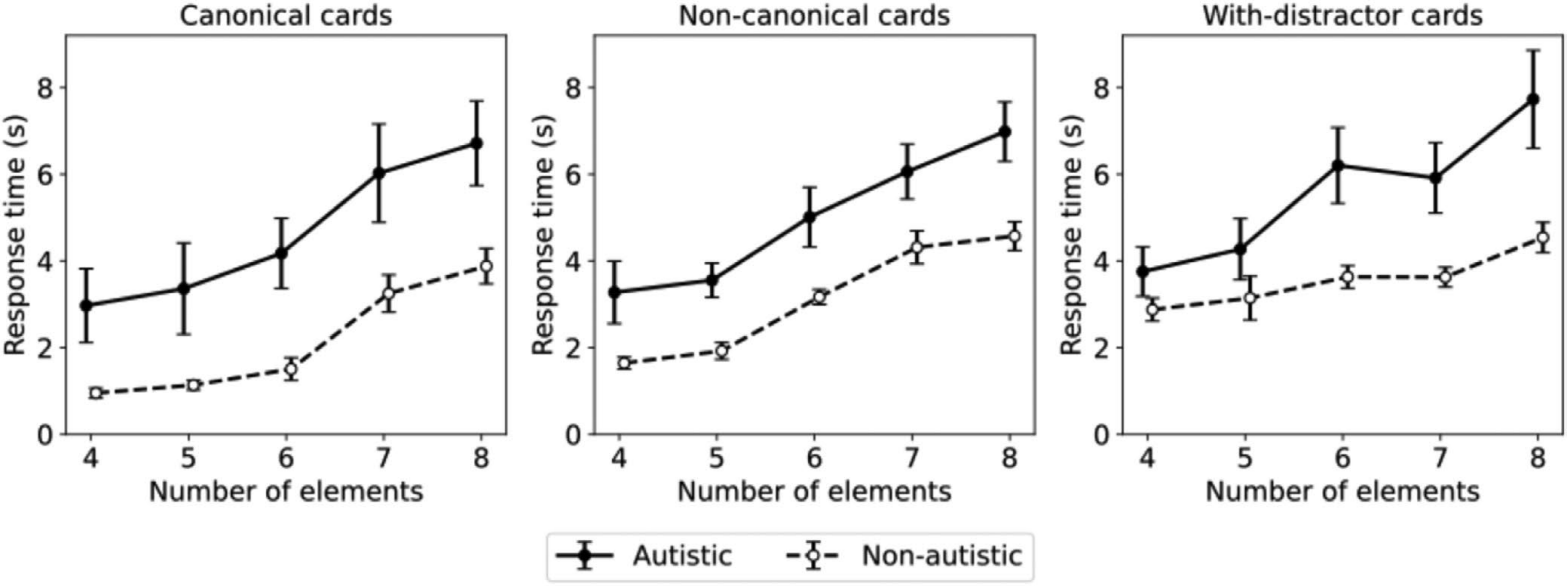
Group of response times for each configuration. Response times for the different series were slightly offset along the x‐axis for visualization purposes.

A more detailed examination of the counting test across different configurations in terms of RT is presented in Figures 1 and 2. Figure 1 shows comparisons between configurations (canonical, non‐canonical and with distractors) within each group, depicting the mean RT of participants for each counting test card, with error bars representing the standard deviation. The visual comparison was corroborated by linear mixed‐effects models including centered age, centered number of elements to be counted, configuration and the interactions between number of elements and configurations as fixed effects. The number of elements had a significant effect in both groups (β=1.03, SE = 0.19, p<0.001 in the autistic group; β=0.80, SE = 0.09, p<0.001 in the non‐autistic group). Regarding configuration effects, both non‐canonical and with‐distractor arrangements differed significantly from the canonical condition in non‐autistic participants (β=0.98, SE = 0.17, p<0.001; and β=1.41, SE = 0.17, p<0.001, respectively). As shown in the right panel of Figure 1, RTs were consistently higher for non‐canonical than for canonical arrangements, and counting with distractors typically required more time when the number of elements was fewer than six. In contrast, in the autistic group, the effect of non‐canonical configurations was not significant, whereas the effect of distractor configurations showed weaker statistical evidence (β=0.94, SE = 0.37, p=0.011), possibly reflecting greater variability in RT (see error bars in the left panel of Figure 1). The interaction between number of elements and configuration was not significant, indicating that the shape of the RT functions across increasing set sizes did not differ between configurations, with the exception of the distractor configuration in the non‐autistic group (β=1.41, SE = 0.17, p<0.001). The effect of age, not depicted in Figure 1, was significant in both groups, with RT decreasing with age (β=−1.56, SE = 0.55, p=0.004, in the autistic group; and β=−0.52, SE = 0.11, p<0.001, in the non‐autistic group).

Moreover, differences in RT between canonical and non‐canonical arrangements at the individual participant level were highly variable, even in the non‐autistic group, despite the fact that the group mean clearly distinguished the two configurations for quantities less than six, making it impossible to replicate the findings reported by Jarrold and Russell (1997).

Figure 2 compares groups within each configuration. Visual inspection was complemented by linear mixed‐effects models including group, centered age, centered number of elements to be counted, and the interaction between group and number of elements as fixed effects. Significant differences between groups were observed in all configurations (β=2.62, SE = 0.79, p=0.001, in canonical; β=1.91, SE = 0.48, p<0.001, in non‐canonical; and β=2.04, SE = 0.63, p=0.001, in with‐distractor arrangements). Age and number of elements also significantly influenced RT in all configurations. The interaction between group and number of elements was significant only when counting with distractors (β=0.57, SE = 0.21, p=0.006), indicating that RT curves showed a similar overall shape in both groups for canonical and non‐canonical arrangements, whereas the rate of increase in RT with increasing number of elements was greater in autistic participants when counting with distractors.

Regarding the modal strategy, VCP was the most frequently observed among autistic participants (*n* = 28), compared with non‐autistic participants (*n* = 7). In contrast, N was more frequently observed in non‐autistic participants (*n* = 32) than in autistic participants (*n* = 13). Strategies P and VC were each the modal strategy of one non‐autistic participant, whereas none of the autistic participants showed these as their modal strategy. Using the modal strategy as a reference is quite representative, as 20 participants in the autistic group and 18 participants in the non‐autistic group employed the same strategy across all cards of the counting test. Among these, 16 autistic participants used VCP and 4 used N, whereas in the non‐autistic group 2 used VCP and 16 used N.

Binary logistic regression analyses with interaction were subsequently conducted to examine whether group membership predicted the use of specific strategies while controlling for centered age. Separate models were fitted for VCP and N (each contrasted against all remaining strategies). Results are presented in Table 2 as odds ratios with 95% confidence intervals, obtained by exponentiating the estimated regression coefficients. The analyses suggested that autistic participants showed a significantly higher likelihood of using VCP as their modal strategy and a significantly lower likelihood of using N compared with non‐autistic participants. Age was also a significant predictor in both models, with younger participants being more likely to use VCP and older participants more likely to use N. However, the interaction between group and age was not statistically significant in either model.

**TABLE 2:** Group comparison of frequencies for the modal strategy across the counting test cards.

	VCP			N		
	Odds ratio	95% CI	*p*	Odds ratio	95% CI	*p*
Group (ASD)	41.20	[6.32, 268.54]	< 0.001***	0.04	[0.01, 0.21]	< 0.001***
Age	0.12	[0.02, 0.62]	0.011*	7.31	[1.72, 31.03]	0.007**
Group × age:	2.80	[0.45, 17.51]	0.271	0.40	[0.08, 2.16]	0.289

*Note:* *< 0.05, **< 0.01, ***≤ 0.001.

**FIGURE 3:**
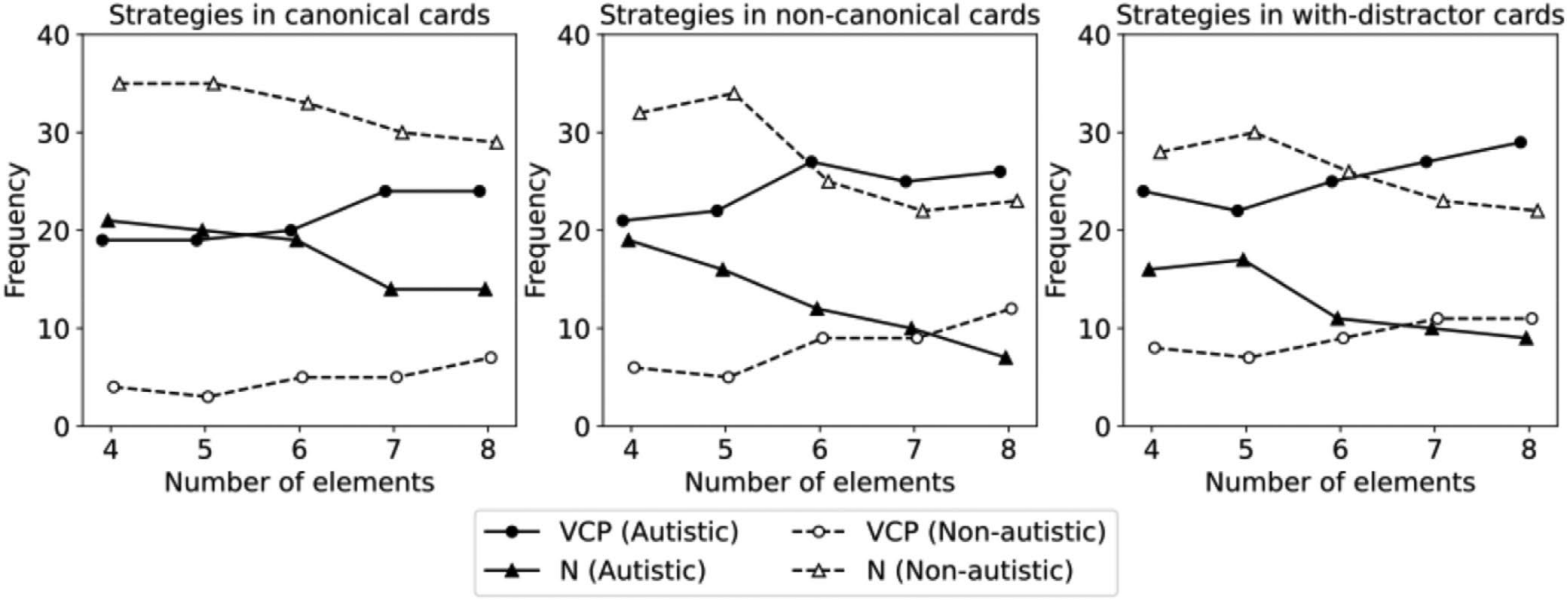
Frequencies for VCP and N strategies for each counting test card. Frequencies for the different series were slightly offset along the x‐axis for visualization purposes.

Frequencies for VCP and N strategies are shown for each counting test card in Figure 3. The autistic group used both strategies for small quantities in canonical configurations but relied more heavily on the VCP strategy when counting quantities above six. They also tended to use VCP more frequently in non‐canonical configurations and, particularly, in presence of distractors, even for small quantities. In contrast, the non‐autistic group relied primarily on the N strategy across all configurations. However, a slight increase in VCP usage was also observed for non‐canonical and with‐distractor arrangements. Additionally, the overall shape across counting quantities is similar for both groups, suggesting that some participants used VCP as the number of elements increased. Age effects are not expected to confound this comparison, as the groups were age‐matched and, as shown in Table 2, the effect of age did not appear to differ significantly between autistic and non‐autistic participants.

Before addressing the third research question, Spearman's correlation tests were conducted to examine the relationship between age and the other measures within each group. In the autistic group, age was significantly correlated with conservation, RT, and modal strategy (ρ=0.34, p=0.030; ρ=−0.52, p<0.001; and ρ=0,42, p=0.006, respectively), but not with accuracy or cardinality; although the latter showed a trend‐level association (ρ=0.30, p=0.056). In the non‐autistic group, age was significantly correlated with all measures, with coefficients in absolute value ranging from 0.50 to 0.69 and *p*‐values below 0.001.

Table 3 presents Spearman's partial correlation coefficients (controlling for age) between the modal strategy and cardinality, conservation, and the counting test variables of accuracy and RT averaged across counting test cards. In the autistic group, correlations with the modal strategy were moderate and statistically significant across all measures, whereas no significant correlations were found in the non‐autistic group after removing the effect attributable to age.

**TABLE 3:** Spearmans partial correlation coefficients between the modal strategy and cardinality, conservation, and accuracy and response time averaged across counting test cards, controlling for age.

	Autistic		Non‐autistic	
	*ρ*	*p*	*ρ*	*p*
Accuracy	0.363	0.021*	0.009	0.956
Response time	−0.444	0.004**	−0.114	0.484
Cardinality	0.385	0.014*	0.070	0.666
Conservation	0.408	0.009**	0.306	0.055

*Note:* *< 0.05, **< 0.01, ***≤ 0.001.

The results of the ANCOVA analyses are presented in Tables 4 and 5. Given the very low representation of the P and VC strategies in the sample, a binary variable was created for the analyses, coded as 1 when the modal strategy was N and 0 otherwise. All models reached statistical significance except for accuracy averaged across counting test cards in the autistic group (F=2.48,p=0.098). The effect of preferring the N strategy over other strategies was significant only for the conservation score within the autistic group and was close to reaching significance for accuracy. The low adjusted *R* ^2^ values (ranging from 0.07 to 0.19 in the autistic group and from 0.23 to 0.46 in the non‐autistic group) indicated that strategy preference accounted for only a small proportion of the variability in these measures. Age was not a significant covariate in any of the models for the autistic group. In contrast, age emerged as a significant covariate in all models for the non‐autistic group, which also showed higher adjusted *R* ^2^ values.

**TABLE 4:** Impact of the strategy on the accuracy, response time, cardinality, and conservation scores in the autistic group.

		(SE)	*p*	95% CI
Accuracy	Intercept:	0.82 (0.04)	< 0.001***	[0.75, 0.89]
	N:	0.14 (0.07)	0.056	[−0.00, 0.28]
	Age:	0.00 (0.03)	0.931	[−0.06, 0.07]
Response time	Intercept:	5.86 (0.68)	< 0.001***	[4.48, 7.25]
	N:	−2.09 (1.30)	0.115	[−4.72, 0.53]
	Age:	−1.16 (0.59)	0.058	[−2.35, 0.04]
Cardinality	Intercept:	0.85 (0.04)	< 0.001***	[0.78, 0.93]
	N:	0.12 (0.07)	0.101	[−0.02, 0.26]
	Age:	0.03 (0.03)	0.289	[−0.03, 0.10]
Conservation	Intercept:	0.63 (0.06)	< 0.001***	[0.50, 0.76]
	N:	0.29 (0.12)	0.020*	[0.05, 0.54]
	Age:	0.04 (0.06)	0.494	[−0.07, 0.15]

*Note:* *< 0.05, **< 0.01, ***≤0.001.

**TABLE 5:** Impact of the strategy on the accuracy, response time, cardinality, and conservation scores in the non‐autistic group.

		(SE)	*p*	95% CI
Accuracy	Intercept:	0.88 (0.05)	< 0.001***	[0.78, 0.99]
	N:	0.02 (0.06)	0.760	[−0.10, 0.14]
	Age:	0.09 (0.03)	0.001***	[0.04, 0.14]
Response time	Intercept:	3.10 (0.28)	< 0.001***	[2.54, 3.66]
	N:	−0.21 (0.32)	0.528	[−0.86, 0.45]
	Age:	−0.47 (0.13)	0.001**	[−0.74, −0.20]
Cardinality	Intercept:	0.90 (0.05)	< 0.001***	[0.79, 1.01]
	N:	0.05 (0.07)	0.403	[−0.08, 0.18]
	Age:	0.07 (0.03)	0.015*	[0.01, 0.12]
Conservation	Intercept:	0.82 (0.06)	< 0.001***	[0.70, 0.93]
	N:	0.11 (0.07)	0.104	[−0.02, 0.25]
	Age:	0.11 (0.03)	0.001***	[0.05, 0.16]

*Note:* *< 0.05, **< 0.01, ***≤ 0.001.

## 4 Discussion

This study addresses the gap in understanding how cardinality and numerical conservation develop and relate to counting strategies in autistic and non‐autistic children. To investigate this, we compared both groups (*n* = 41 each; aged 4–7) using measures of cardinality and conservation, together with an adapted counting task assessing counting strategies and performance (accuracy and response times).

### 4.1 Group Differences

Our first research objective was to examine the understanding of cardinality and conservation in children aged 4–7 years with and without autism. We did not observe differences in the understanding of cardinality, but there were differences with respect to conservation, which was tested in three ways: (1) reproducing the arrangement of one to four elements demonstrated by the examiner, (2) modifying sets of objects so that the total matches a specified sum or difference, and (3) reporting the number of objects in a set after the arrangement has been altered. (1) and (2) test equinumerosity, while (3) tests proper Piagetian conservation. As will be explained, these differences may relate to the preference observed in autistic children for the VCP strategy (verbal counting with pointing), which is arguably less efficient in these conservation tasks.

The second aim was to investigate potential differences between the autistic and non‐autistic groups in counting‐related variables, including response time, success rates, and counting strategies. We observed that autistic children made heavier use of VCP than their non‐autistic peers, without this affecting accuracy in counting tasks. When comparing groups, although a shift in frequencies is evident, the overall shape of the corresponding curves remains similar. This suggests that the effect of increasing the number of items to be counted (a potential change of strategy) is similar for children in both groups. In the autistic group, more children used the VCP strategy than the N strategy, except when counting small quantities (< 6) in canonical arrangements. However, half of the autistic group used the VCP strategy also for counting less than six elements arranged canonically, suggesting a high overall reliance on VCP at the expense of N.

Autistic children were slower than non‐autistic children in counting canonical, non‐canonical, and with‐distractors displays. Additionally, as shown in Figure 1, non‐autistic children appear to benefit more clearly from having canonical arrangements than autistic children, a result consistent with Jarrold and Russell (1997), although the variability in our data limits the clarity of this effect. In non‐autistic participants, RTs are longer in the non‐canonical condition than in the canonical condition, indicating a processing cost associated with non‐canonical presentations. This cost becomes even more pronounced in the presence of distractors, where RTs increase further. TD participants require more time in the distractor condition than in the non‐canonical condition, particularly for quantities smaller than six. In autistic participants, performance is characterized by greater variability. Unlike the pattern observed in non‐autistic participants, there are no significant differences between the canonical and non‐canonical conditions. However, a significant difference does emerge when comparing the canonical condition to the distractor condition, suggesting that the presence of distractors disproportionately affects RTs in this group (see also Figure 2). The observed general group difference in RTs cannot relate just to autistic children's preference for VCP, given that autistic children exhibited longer RTs even when they employed the N strategy. Thus, it is unlikely that the differences in RTs are only related to motor coordination, since motor coordination is not involved in N, suggesting instead cognitive differences – ranging from executive functions to proper mathematical skills.

Overall, autistic children appear to be just as accurate as their non‐autistic peers in counting and in understanding the cardinality principle, even though they are likely to subitize less than their non‐autistic peers. It is important to clarify this point (which we discuss in more detail below). We do not have direct evidence of subitizing in any of the groups. However, we believe it is still reasonable to suspect that autistic children engaged in subitizing less often than TD children. The reasoning behind this claim is the following: autistic children engaged in N less often than TD children. We believe it is reasonable to assume that, given the age range, at least several instances of N involved subitization, though N is compatible with internal counting. If this assumption holds, it follows that autistic children may have engaged in subitizing less frequently than their TD peers, as they used N less often.

### 4.2 Counting Strategies

With respect to the third research objective—to explore which counting strategies correlate with better performance and faster response times on the counting test, as well as with mastery of cardinality and conservation‐, both VCP and N seem to be just as effective for supporting the cardinality principle as other strategies (or even switching flexibly among several strategies).

With respect to conservation, however, the picture seems different. Whereas VCP is well suited for determining how many items are present in a single set, number conservation tasks place additional demands on the child, which go beyond merely enumerating items. Conservation requires recognizing that the numerosity of a set remains unchanged despite transformations. To make this judgment, children must do more than recite the count sequence or coordinate pointing with verbal labels. They need to compare two sets or two successive states of the same set, often under conditions where perceptual cues are misleading (Piaget 1952).

These comparisons appear to rely on forms of reasoning that are not automatically supported by verbal counting. For example, conservation may require mentally representing both sets simultaneously and coordinating these representations to determine numerical equivalence. Such processes may call for non‐verbal or conceptual strategies, such as tracking one‐to‐one correspondences, maintaining a stable mental model of quantity, or relying on an internalized sense of equivalence, rather than simply recounting items. In this sense, conservation tasks seem to draw on a broader range of cognitive tools than those mobilized during ordinary counting. Among these, executive functions play a major role, which are well documented as areas of difficulty in autism (Hill 2004; Demetriou et al. 2018). Furthermore, perceptual cues, which, as said, are misleading in conservation tasks, may be processed atypically in autism, in line with other perceptual atypicalities (Mottron et al. 2006; Happé and Frith 2006).

Thus, while consistent verbal counting strategies may be perfectly adequate for supporting cardinality, they do not necessarily equip children to meet the demands of conservation, which appears to require comparing sets in ways that cannot be achieved through verbal counting and pointing alone. In the autistic group we found a relation between the use of N and conservation that was absent in the TD group – where success in conservation related to age. As said, we cannot know if N involves subitizing or internal counting. However, in either case, N involves using internal representations (see Wender and Rothkegel 2000 for different accounts of subitization). It is suggestive to think that, at least in the case of autistic children, succeeding in conservation tasks required this ability to recruit internal representations.

### 4.3 Developmental Effects

When considering the full sample of 82 children, age shows a significant effect on cardinality, conservation, and success scores, with older children systematically achieving higher performance. In contrast, age does not have a significant effect on RTs. There is also an indication that the effect of age may differ between the autistic and the non‐autistic groups for success and RT, although these interaction effects do not reach statistical significance and should therefore be interpreted cautiously. No such differential age‐related effect is observed for cardinality or conservation. Age also influences the use of strategies. Younger children are more likely to rely on VCP strategies, whereas older children tend to use N‐based strategies. This age‐related shift in strategy use does not differ significantly between the autistic and the non‐autistic groups.

The patterns observed might suggest potential differences in developmental trajectories between groups, though it is more likely that the observed patterns are related to heterogeneity in the autistic group. Success appears to increase more steeply with age in the non‐autistic group than in the autistic group, where we see autistic children across all ages who continue to exhibit relatively low success scores. As said, when considering the full sample of children and examining mean RTs, RT does not appear to vary significantly with age, likely due to the substantial variability present in the data. However, a more fine‐grained analysis reveals a different pattern: when RT is examined at the item level within each group, it does in fact decrease with age in both groups. Importantly, the magnitude of this age‐related decrease differs between groups. The coefficient for the autistic group (−1.56) is larger in absolute value than that for the non‐autistic group (−0.52), indicating a steeper decline in RT with increasing age among autistic participants. This pattern is consistent with the group comparisons described earlier, reinforcing the conclusion that age‐related improvements in processing speed are more pronounced in the autistic group, despite their overall higher RTs across all ages. This effect appears to be driven by particularly high RTs among younger autistic children.

In sum, in the non‐autistic group, age is significantly correlated with all measured variables: success, mean RT, cardinality, conservation, and preferred strategy. In contrast, in the autistic group, age is significantly correlated only with mean RT, preferred strategy, and conservation (although the effect of age on conservation was no longer significant after controlling for the use of the N strategy), but not with success or cardinality. As mentioned, we think these results reflect heterogeneous developmental paths in autism.

### 4.4 Comparison With Other Studies

There is extensive research in typical development examining the understanding of fundamental principles such as cardinality and conservation (e.g., Sarnecka and Wright 2013), as well as their relationship with counting strategies (e.g., Jacobs et al. 2021, Flowers et al. 2019). However, studies examining how autistic children acquire these principles are scarce (e.g., Titeca et al. 2014, 2015, 2017), and none of them provides an in‐depth or detailed account of the relationships between them. On the other hand, the few existing studies examining these principles in autistic children throw mixed results. For instance, Titeca et al. (2017) report no differences in basic numerical skills (e.g., counting or subitizing) between 4‐ and 5‐year‐old autistic and non‐autistic children, while Jarrold and Russell (1997) observed that autistic individuals with a verbal mental age between 4 and 9 years tended to rely more on “analytical counting” than on subitizing, suggesting some difficulties in the latter strategy ‐although Jarrold and Russell did not find inter‐group significant differences in terms of how many individuals in each group employed one strategy or the other.

In line with Jarrold and Russell's ‐ and contrary to Titeca et al.'s‐ findings, our interpretation of the results indicates that autistic children may experience more difficulties with subitizing. However, this apparent contradiction is neither straightforward nor direct. Titeca et al. (2017) assessed accuracy in brief‐exposure subitizing tasks, whereas we did not measure subitizing directly. In our counting tasks, children were free to use whatever strategy they preferred. Moreover, our category N does not map cleanly onto subitizing, since, as noted several times, children could, in principle, have been counting using inner speech. We observed that autistic children used N less frequently than non‐autistic children, relying more on VCP than their TD peers, and therefore, probably engaging less in subitization, under the assumption that at least on some occasions, N does involve subitizing. As indicated, this would suggest that they find subitization more difficult than non‐autistic children. At any rate, autistic children seem to exhibit some difficulties with using internal representations in counting tasks. Still, one might wonder whether autistic children simply prefer VCP over N, rather than finding N difficult. As mentioned above, it may be that they tend to use VCP more for reasons related to insistence on sameness or uncertainty. Perhaps if required to use N, their accuracy could match that of non‐autistic children. This is yet another reason why we find it difficult to compare our results with Titeca et al.'s (2017). In sum: on the one hand, the N strategy may or may not correspond to subitization; on the other, we don't know if relying more on VCP is the effect of some difficulty or rather reflects preference.

## 5 Conclusions

This study explored how cardinality and numerical conservation relate to counting strategies in autistic and non‐autistic children. Overall, the more notable result of our study concerns performance on conservation tasks: autistic children appear to show difficulties (compared to TDs) in developing conservation abilities, suggesting that several autistic children may already lag behind their peers before encountering formal mathematics instruction (see Fernández‐Cobos et al. 2026; on formal mathematics, see Fernández‐Cobos and Polo‐Blanco 2024). Another interesting result is the lack of difference observed in RTs to canonical and non‐canonical displays in the autistic group, which suggests differences with non‐autistic children in perceptual processing (Mottron et al. 2006). This might also be relevant given that rapid naming of non‐canonical dot‐array stimuli within the subitizing range predicts mathematical performance in TD preschool children (LeFevre et al. 2022; 4–6 years) and school‐aged children (Huotari et al. 2025; grades 3–6).

The hypothesized difficulty with deploying internal representations and the apparent difficulty with conservation may be related, perhaps because both processes rely on shared cognitive mechanisms with which autistic children may struggle. In either case, our findings highlight a potentially important target for early intervention aimed at strengthening foundational mathematical skills in autistic children (e.g., Goñi‐Cervera et al. 2026; Ingelin et al. 2023).

### 5.1 Limitations and Future Research

The study has some limitations. One of them has already been acknowledged: we did not directly measure the subitization ability, as children were not instructed to respond as quickly as possible. This limits direct comparison with previous research focusing on subitizing ability. Secondly, the interpretation of our results in terms of development is unclear. They are suggestive of delay, but, to begin with, the study was not longitudinal but cross‐sectional, so we cannot know if the results really reflect a developmental delay. More importantly, we observed much more heterogeneity in the autistic group than in the non‐autistic group, which points towards heterogeneity in development in the autistic group, rather than to a general delay in that group compared to the non‐autistic group. Thirdly, we cannot determine whether the results observed in the autistic group are attributable to autism as a condition or to a higher prevalence of dyscalculia in the autistic population. Autistic individuals have been found to exhibit greater difficulties than TD peers in mathematics, including early numerical skills (Fernández‐Cobos et al. 2026), suggesting that such difficulties may represent a characteristic of (some subtype of) autism. Nevertheless, the possible contribution of a higher prevalence of co‐occurring dyscalculia cannot be ruled out. Finally, given that no IQ measures were available for the non‐autistic group, we were unable to account for the potential effects of intelligence in the group comparisons, although the IQ distribution in the autistic group did not appear to be atypical.

As a future line of research, and considering our findings on differences in strategy use, it would be interesting to explore the heterogeneity within autistic children according to strategy preference. Such an approach might help identify subgroups with different cognitive profiles and allow for targeted interventions. In particular, interventions to teach subitizing to children who do not spontaneously use it could be developed. Furthermore, given the suggested connection between deploying internal representations and conservation in the autistic group, the impact of these interventions on children's acquisition of conservation could be further examined. This is particularly important given that conservation has been reported to predict mathematical achievement in children with mathematical difficulties (Lambert and Spinath 2018), emphasizing the relevance of fostering this foundational skill in autistic students through early educational interventions.

## Funding

This work was supported by the projects PID2022‐136246NB‐I00, funded by MICIU/AEI and FUNLAT (PID2021‐122233OB‐I00), funded by MICIU/AEI.

## Conflicts of Interest

The authors declare no conflicts of interest.

## Data Availability

The data that support the findings of this study are available on request from the corresponding author. The data are not publicly available due to privacy or ethical restrictions.
